# Determinants and seasonality of major structural birth defects among newborns delivered at primary and referral hospital of East and West Gojjam zones, Northwest Ethiopia 2017–2018: case–control study

**DOI:** 10.1186/s13104-019-4541-4

**Published:** 2019-08-09

**Authors:** Binalfew Tsehay, Desalegn Shitie, Akilog Lake, Erimiyas Abebaw, Amisalu Taye, Enatinesh Essa

**Affiliations:** 1 Department of Biomedical Sciences, Debre Markos University, Debre Markos, Ethiopia; 2 Department of Gynecology and Obstetrics, Debre Markos Referral Hospital, Debre Markos, Ethiopia; 3 Department of Pediatrics, Debre Markos University, Debre Markos, Ethiopia

**Keywords:** Birth defect, Maternal illness, Maternal medication use, Environmental exposure

## Abstract

**Objective:**

Although infant mortality because of birth defect has increased in both developed and developing countries, had not got attention like other health issues at national, regional, or local levels. Documenting the risk factors that influence the occurrence of birth defects and its seasonality will help to inform the community and to develop preventive strategies for the country.

**Results:**

Factors associated with higher likelihood of a major structural birth defects included maternal age; neonates born from women living in urban; and in Dega; history of fever during pregnancy; intake of herbal medicine; and drinking alcohol. Counselling for pregnancy preparation and folic acid supplementation was found protective for the likelihood of birth defect.

## Introduction

Birth defects also are known as, congenital anomalies, congenital disorder or congenital malformations [1, 2] are defined as structural or functional anomalies that occur during intrauterine development and can be identified prenatally, at birth or manifests later in life. The most common major structural birth defects include congenital heart disease, neural tube defects, orofacial clefts, limb reduction defects, and Down syndrome [2].

Every year an estimated 7.9 million children—6 percent of total births worldwide—are born with a serious birth defect of genetic or partially genetic origin. Data presented in a global report on birth defects show that at least 3.3 million children under 5 years of age die from birth defects each year and an estimated 3.2 million of those who survive may be disabled for life. This disability can have a severe human and economic toll on those affected children, their families, communities and health-care systems [3].

Although birth defects are a global problem, their impact is severe in middle- and low-income countries where more than 94% of the births with serious birth defects and 95% of the deaths of these children occur [3]. Furthermore, 15–30% of infant and child hospital admissions are due to birth defects and exact a proportionally higher health care cost than other hospitalizations [4].

Researches on birth defects and associated risk factors were not conducted adequately in Ethiopia and had not got attention like other health issues at national, regional, or local levels.

Documenting the risk factors that influence the occurrence of birth defects and its seasonality will help to inform the community and to develop preventive strategies for the country. Studying the seasonality of congenital anomalies can also help trace risk factors for birth defect.

The main objective of the study was to assess determinants and seasonality of major structural birth defects among newborns delivered at primary and referral hospital of East and West Gojjam zone, Northwest Ethiopia 2017–2018.

## Main text

### Subjects and methods

#### Study design

Institutional based unmatched case–control study design was conducted at selected primary and referral hospital of East and West Gojjam zone from September 2017 to October 2018.

#### Study population

All newborns in selected hospitals who was born from September 2017 to October 2018 and who fulfill the case and control definition criteria for this particular study.

#### Eligibility criteria

*Inclusion criteria* Newborns with major structural birth defect and the next three newborns (delivered after the case) without defect and whose mothers are voluntary, was included in the study.

*Exclusion criteria* Newborns whose mothers could not be interviewed because they were very sick, emotionally upset, and mute or die post-delivery was excluded from the study.

#### Sample size and sampling procedure

The sample size was calculated using the Fleiss with continuity correction factor formula with a case–control ratio of 1:3; assuming the proportion of controls exposed was 50%, the minimum odds ratio to be detected is 2.0, power of 80% and a significance level of 95%. The computed sample size was 398, (100 cases and 298 controls).

#### Variables of the study

*Independent variables* Socio demographic characteristics of mothers and fathers, obstetrical history of mother and newborn, maternal illness and medication use of the mother during pregnancy and, environmental exposures of mothers during pregnancy.

*Dependent variable* Patient control status of major structural birth defect.

#### Data collection procedure

Socio demographic characteristics of mothers and fathers; obstetrical history of mother and newborn; history of maternal illness, medication use, environmental exposures during pregnancy was assessed using structured interviewer administered questionnaire. Patient control status of the fetus/newborn was assessed by detail observation and physical examination.

#### Data analysis

Data was entered via Epi data and analyzed using STATA version 14. Bivariate and multivariate analyses was employed, and factors which had a *p* value of < 0.05 in the bivariate analysis was included in the multivariate analysis. p values of < 0.05 was considered statistically significant.

### Results

A total of 398 newborns, male 219 (55%) and female 179 (45%), with gestational age (using ultrasound) of 16–43 weeks was included in this study. All 398 mothers whose newborns were included, were aged between 18 and 42 years with a mean age of 27.51 years and 388 (97.5%) were married (Table 1).

**Table 1:** Socio demographic characteristics of the study sample

Characteristics	Cases (n = 100)	Controls (n = 298)	Total
Maternal age			
≤ 20	10 (23.8%)	32 (76.2%)	42 (100.0%)
20–35	75 (22.7%)	256 (77.3%)	331 (100.0%)
> 35	15 (60%)	10 (40%)	25 (100.0%)
Marital status of the mother			
Married	94 (24.2%)	294 (75.8%)	388 (100.0%)
Unmarried	6 (60.0%)	4 (40.0%)	10 (100.0%)
Father’s age			
< 35	51 (19.3%)	213 (80.7%)	264 (100.0%)
≥ 35	49 (36.6%)	85 (63.4%)	134 (100.0%)
Residence			
Urban	51 (21.9%)	182 (78.1%)	233 (100.0%)
Rural	49 (29.7%)	116 (70.3%)	165 (100.0%)
Primary address during 1st trimester			
Dega	25 (39.1%)	39 (60.9%)	64 (100.0%)
Woyina Dega	34 (18.6%)	149 (81.4%)	183 (100.0%)
Kolla	41 (27.2%)	110 (72.8%)	151 (100.0%)
Monthly income of the family			
≤ 500	24 (58.5%)	17 (41.5%)	41 (100.0%)
500–10,000	70 (23.3%)	230 (76.7%)	300 (100.0%)
≥ 10,000	6 (10.5%)	51 (89.5%)	57 (100.0%)
Father’s ethnicity			
Amhara	100 (25.3%)	296 (74.7%)	396 (100.0%)
Oromo	0 (0.0%)	2 (100.0%)	2 (100.0%)
Mother’s ethnicity			
Amhara	96 (25.1%)	286 (74.9%)	382 (100.0%)
Oromo	0 (0.0%)	8 (100.0%)	8 (100.0%)
Tigrie	4 (50.0%)	4 (50.0%)	8 (100.0%)

In this study, most of the major structural birth defects occur during spring and least occur during autumn. But Chi square test of seasonal patterns of birth defects shows that there is no statistically significant association between season of birth- and absence or presence of birth defects (p = 1.00).

#### Distributions of major structural birth defects among cases

**Fig. 1:**
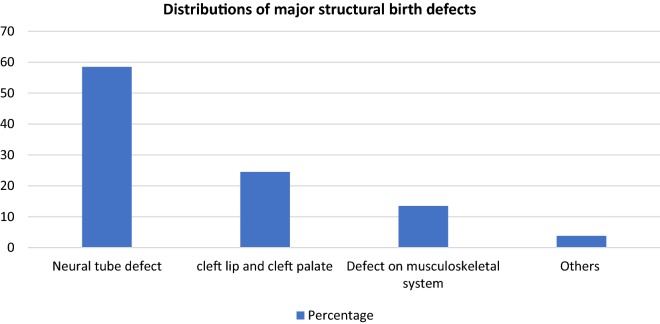
Distributions of major structural birth defects among cases

During the study period, 100 new born were found with major structural birth defects in a proportion as depicted on Fig. 1.

#### Determinant factors associated with major structural birth defects

Socio-demographic characteristics of mothers and fathers; the obstetrical history of the mother and newborn; the history of maternal illness, medication use, environmental exposures during pregnancy were analyzed in bivariate and multivariate logistic regression. We identified risk and protective factors for the occurrence of the major structural birth defects as depicted by Table 2.

**Table 2:** Risk and protective factors associated with major structural birth defects

Characteristics	Cases	Controls	COR with 95% CI	AOR with 95% CI
	Count (%)	Count (%)		
Maternal age				
≤ 20	10 (10%)	32 (10.7%)	1.1 (0.5–2.2)	
20–35	75 (75%)	256 (85.9%)	1	1
> 35	15 (15.0%)	10 (3.4%)	5 (2.2–11.9)	4.9 (1.1–23.7)
Residence				
Urban	51 (21.9%)	182 (78.1%)	0.7 (0.4–1.2)	6.4 (1.9–21.7)
Rural	49 (29.7%)	116 (70.3%)	1	1
Primary address during 1st trimester				
Dega	25 (39.1%)	39 (60.9%)	2.8 (1.5–5.2)	4.3 (1.3–14)
Woyina Dega	34 (18.6%)	149 (81.4%)	1	1
Kolla	41 (27.2%)	110 (72.8%)	1.6 (1.0–2.7)	
Intake of herbal medicine during pregnancy				
Yes	40 (40%)	36 (12%)	4.9 (2.9–8.2)	10.9 (4.2–28.1)
No	60 (60%)	262 (88%)	1	1
Alcohol intake				
Yes	92 (92%)	164 (55%)	9.4 (4.4–20)	12.7 (3.3–48.7)
No	8 (8%)	134 (45%)	1	1
History of high fever during pregnancy				
Yes	54 (54%)	48 (16%)	6 (3.7–10.0)	3.4 (1.3–11.6)
No	46 (46%)	250 (84%)	1	1
Folic acid				
Yes	31 (31%)	228 (76.5%)	1	1
No	69 (69%)	70 (23.5%)	7.2 (4.39–12.0)	7.3 (2.9–18.8)
Counselling for pregnancy preparation				
Yes	30	190	1	1
No	70	108	4 (2.5–6.7)	4.8 (1.9–12.1)

#### Risk factors of major structural birth defects

Factors associated with higher likelihood of a major structural birth defects included maternal age (AOR: 4.9, 95% CI 1.1–23.68); neonates born from women living in urban (AOR: 6.4, 95% CI 1.9–21.7); history of fever during pregnancy (AOR: 3.4, 95% CI 1.3–11.6); intake of herbal medicine(AOR: 10.9, 95% CI 4.2–28.1); and drinking alcohol (AOR: 12.7, 95% CI 3.3–48.7) (Table 2).

#### Protective factors of major structural birth defects

Counselling for pregnancy preparation (AOR: 4.8, 95% CI 1.9–12.1) and folic acid supplementation (AOR: 7.3, 95% CI 2.9–18.8) was found protective for the likelihood of birth defect (Table 2).

### Discussion

#### Risk factors for the incidence of major structural birth defects

Many studies have been conducted to determine the association of various risk factors with the incidence of birth defects. For example, in Kenya, Tanzania and Iran the number of malformed babies appeared to increase with increasing maternal age especially from 35 years and above [5–7]. The same is true in our study; Maternal age was significantly associated with birth defect. Women above 35 years old were around five times more likely to have neonates with birth defect as compared to those women who are in the age group of 20–35 years old. However, a research done in Addis Ababa and Saudi Arabia [8, 9] showed that there is no significance difference between cases and controls regarding maternal age for occurrence of birth defects.

In rural areas of Gabon structural birth defects were rare or absent as compared to the number recorded in urban areas [10]. In this study neonates born from women living in an urban area were about six times more likely to develop birth defect as compared to those neonates born from women who live in a rural setting and the odds of women to have a newborn with the birth defect was around four times higher among women living in Dega as compared to women whose primary address was in Woyina Dega. This was supported by research on epidemiology of birth defects based on a birth defect surveillance system from 2005 to 2014 in Hunan province, China [11].

This may be due to diet diversification habits and non-fat diet in rural areas of Ethiopia as has been shown its effect in an animal model study [12] and a greater level of air pollution in urban areas [13].

Studies conducted in a variety of settings around the world have observed a significant association between maternal fever and birth defects consistent with our findings [6, 14, 15]. A population-based case control study done in Shanxi province northern China on risk factors for neural tube defects found that a history of fever during the periconceptional period was associated with almost a threefold increase in risk for neural tube defects, which persisted even after controlling for other covariates [16, 17]. This were in congruent with our study on which mothers who had history of fever during pregnancy were around three times more likely to have neonates with birth defect even after adjusting for other factors.

As reported from northern Ghana the use of herbal medicines by pregnant women poses a potential danger to the fetus and development of 70% birth defects of unknown etiology [18]. In our study intake of herbal medicine during pregnancy was significantly associated with major structural birth defects. Those women who took herbal medicine during pregnancy were around eleven times more likely to get a new born suffering from birth defect compared to women who didn’t take herbal medicine while pregnant. In Ethiopia pregnant women used herbal medicine in the first trimester of pregnancy [19] in which organogenesis and birth defect occur [20].

The 2011 Ethiopia Demographic and Health Survey found that 45% of women and 53% of men reported drinking alcohol at some point in their lives [21]. The association of alcohol drinking during and before early pregnancy and birth defect was reported as having significant association [8, 22, 23].

In our study Maternal history of alcohol intake during pregnancy was found to be significantly associated with birth defect. Women who took alcohol during their pregnancy were around thirteen times more likely to have newborns with birth defect as compared to those women who didn’t take alcohol during their pregnancy.

Both moderate and high levels of alcohol intake during early pregnancy may result in alterations in growth and morphogenesis of the fetus. Microcephaly, short palpebral fissures, epicanthal folds, maxillary hypoplasia, short nose, thin upper lip, abnormal palmar creases, joint anomalies, and congenital heart disease are also present in most infants [1].

#### Protective factors for the incidence of major structural birth defects

Counselling has a positive effect on a range of health outcomes like prevention of birth defects [24]. Physician counseling can reduce risk of medication-induced birth defects [25] and increase intake of folic acid before conception [26] so can reduce incidence of birth defect as explained by many researches.

Similar to above report counselling for pregnancy preparation in our study was found protective for the incidence of birth defect. Women who didn’t get counselling for pregnancy preparation were about five times more likely to have neonates with the birth defect as compared to women who got counselling for pregnancy preparation.

Nearly one-half of pregnancies are unintended so preconception care should be considered an integral part of primary care for women of reproductive age. Provide counselling regarding common issues in preconception care like family planning, screening and treatment for infectious diseases, updating appropriate immunizations, and reviewing medications for teratogenic effects can prevent poor birth outcomes including birth defect [27].

Researches in different setup showed that women who took folic acid were less likely to have babies with birth defects as compared to those who did not take folic acid during and before early pregnancy [6, 28–34].

Intake of folic acid supplementation can reduces nearly 75% of the rate of neural tube defects [35]. It is recommended to take 4 to 5 mg of folic acid daily starting 3 months before conception and continuing until 12 weeks post conception [36, 37].

In our study women who didn’t get folic acid supplementation at or before pregnancy were about seven times more likely to have neonates with birth defect as compared to those women who had been supplemented with folic acid during and before pregnancy which is consistent with other studies that have shown folic acid supplementation is protective against birth defects [37–41].

Folate acts as a cofactor for enzymes involved in DNA and RNA biosynthesis. Interruption of DNA biosynthesis or methylation reactions could prevent the proper closure of the neural tube. Such inhibition could be caused by simple deficiency of either folic acid or vitamin B12 [42].

## Limitations

In this study, there is no statistically significant association between season of birth and the absence or presence of birth defects. But the data should be collected and analyzed based on the season of conception rather than the season of birth or termination. This was the main limitation of the study.

## Data Availability

The dataset supporting the conclusion of this article is available from the authors on request.

## Abbreviations

AOR: adjusted odds ratio
CI: confidence interval
